# Meetings of Societies

**Published:** 1909-06

**Authors:** 


### MEETINGS OF SOCIETIES.

### Bristol Medico-Chirurgical Society.

*March* 10*th*, 1909.

Dr. J. MICHELL CLARKE, President, in the Chair.

LT.-COL. A. W. PRICHARD, V.D., made a statement regarding the 3rd South Midland Field Ambulance (R.A.M.C., T.F.). This new unit, which had been authorised by the War Office about twelve months previously, had been raised in Bristol, and had recruited so successfully that the establishment was now complete as regards both officers and rank and file. A suggestion was made that the medical profession of the city should combine to make some presentation to the newly-raised medical corps as a mark of appreciation of its successful establishment. Resolution : " That the details of the suggestion be left in the hands of a sub-committee consisting of Dr. S. Smith, Mr. Cross and the Secretary, who shall collect subscriptions for the purpose indicated."

Dr. H. P. Hawkins, of London, gave an address on the **Natural History of Ulcerative Colitis and its bearing on treatment.** After dealing with the pedigree of the disease, which can be traced back for nearly 300 years as epidemic dysentery or " bloody flux," Dr. Hawkins dealt with the gradual decline of the disease in epidemic form in England until the sporadic cases ceased to attract much attention, until they eventually reappeared under the new name of " Ulcerative Colitis." The various forms of the disease were described : (*a*) the acute continuous disease ; (*b*) the chronic disease, continuous and intermittent ; (*c*) dysenteric diarrhœa ; (*d*) the acute and the chronic disease ; (*e*) the hemorrhagic disease (lower segment type). The variations did not necessarily betoken separate forms of disease. From a consideration of the bacteriological and clinical evidence, there were grounds for thinking that our long-continued cases may be related to the short fatal cases, both perhaps owing their origin to one of the organisms of the dysenteric group (Flexner and Shiga). The chronic British disease may be a secondary non-specific ulceration, in which case the prospect of a generally applicable specific treatment becomes remote ; though the proved value of serum treatment in tropical bacillary dysentery made one hope that the British disease was the very type which should yield to serum or vaccine treatment. Treatment with Shiga-Flexner serum and with coli vaccine had not yielded very marked success. Shiga's calomel treatment had appeared the best line to adopt ; right-sided colostomy with irrigation of the large bowel was, in his opinion, likely to prove the most life-saving proceeding in the long run. It affords complete rest to the colon, as well as efficient irrigation. A discussion followed, in which Dr. Shingleton Smith, Dr. Walker Hall, Mr. Hey Groves, Dr. Symes, Dr. Every-Clayton, Mr. Stack, Dr. Cary Coombs, Mr. Bush, Mr. Lace and the President took part.

---

*April, 14th,* 1909.

Dr. J. Michell Clarke, President, in the Chair.

Mr. Munro Smith read a paper on **Rupture of the Plantaris Tendon.** Case 1 : A man of sixty, when out shooting, felt a sudden pain in his calf, as though struck with a stone or stick. He hobbled home with difficulty. When seen by the speaker there was no external sign of injury visible, but there was a tender spot on the inner side of the leg half-way down. He was told to rest the limb. Two or three days later there was some œdema over the front of the tibia. He went to business, and would not have any fixed apparatus applied, and disability continued. Later he went to a bone-setter, and a heavy plaster case was applied and worn for fourteen days. The man walked with a limp for six months. Case 2 was that of an athletic man who, when on the point of diving from a diving board, felt that something had struck him on the calf. He looked round to see what it was. On leaving the bath the man found that he was lame. When seen by the speaker seven weeks later, he also had a tender place half-way down the calf, and œdema of the front of the leg. The literature on the subject was very scanty, and the cases mentioned were the only ones the speaker had seen.—Dr. JAMES SWAIN related his personal experiences of a similar accident, which occurred when he was playing tennis. The sudden pain was like that of a whip stroke. He reached home with difficulty. He had strapping applied from the ankle to the knee, and this gave great relief immediately. No pain was felt after three or four days, and at the end of a week he thought all was well, and had the strapping removed. Then pain returned, and the strapping had to be re-applied. He was well in three weeks. The best treatment was, he believed, to strap the limb and allow movement, not to rest the limb in splints.—Dr. A. R. SHORT mentioned a case where symptoms very similar to those mentioned above had been due to thrombosis in a vein of the leg.

Dr. CECIL WILLIAMS showed a case of **Joint Swellings associated with enlargement of lymphatic glands,** and with the development of pes cavus. The patient was a boy who had been admitted to hospital for pain and swelling in the knees. At first there were no glandular swellings, but these developed later. Shortly before the glandular affection there was an elevation of temperature and sponginess of the gums, but these troubles had been transient. Lastly pes cavus on the left side became noticeable. The speaker thought the case was one of Still's disease. In skiagrams the knee-joints appeared normal. There was no reason to think the boy had had either gonorrhœa or syphilis, and Calmette's reaction was not obtainable.—Dr. EDGEWORTH remarked that the case was rather puzzling in that pes cavus had been acquired with extensor plantar reflex. What had this condition to do with the knee trouble ? The general view was that pes cavus of this type was due to some pyramidal-tract affection together with myotatic irritability. Possibly both the knee and foot condition was due to some nerve lesion, but the tenderness of the knee was against that view.—THE PRESIDENT remarked that trophic joint troubles sometimes were of a sub-acute character, and in one case he had seen the joint, which was tender and painful, looked as though pus would have to be evacuated, though it contained sterile fluid.

Dr. RENDLE SHORT gave a demonstration of some **Parasites of Tropical Diseases.**

Dr. ALEXANDER read a paper on **Cretinism and the Puerperium: a Sequel to Thyroid Treatment.** (Reported in full, *vide* p. 128).

Dr. WATSON WILLIAMS read a note on **Two Radical Mastoid Operations** with features of interest, and on some **Points in the Technique of the Radical Operation** for chronic otitis media purulenta. A case was shown proving how good the hearing power might be after the operation, for the patient could hear a loud whisper at a distance of seven feet. The speaker had used the periosteum of the mastoid to line the bony cavity which was made. He thought it was a good thing in some cases to leave the mucous membrane intact, especially near the foramen ovale. In other sinuses good results followed when mucous membrane was left intact.

---

*May 12th*, 1909.

Dr. J. MICHELL CLARKE, President, in the Chair.

The meeting was devoted to a discussion on **Treatment by Ionisation or Cataphoresis.** (*Vide* p. 132.)

J. LACY FIRTH.
J. A. NIXON, *Hon. Sec.*

---

